# Comparing myopic error in patients with basic and convergence insufficiency intermittent exotropia in China

**DOI:** 10.1186/s12886-023-03043-8

**Published:** 2023-06-26

**Authors:** Qingyu Meng, Lejin Wang, Mingwei Zhao, Xi Wu, Lili Guo

**Affiliations:** 1 Department of Ophthalmology & Clinical Centre of Optometry, Peking University People’s Hospital, Beijing, China; 2 Eye Disease and Optometry Institute, Peking University People’s Hospital, Beijing, China; 3 Beijing Key Laboratory of Diagnosis and Therapy of Retinal and Choroid Diseases, Beijing, China; 4 College of Optometry, Peking University Health Science Center, Beijing, China

**Keywords:** Intermittent exotropia, Myopia, Ocular dominance, Convergence insufficiency, Anisometropia

## Abstract

**Purpose:**

To compare the degree of myopia between the dominant and non-dominant eyes in teenagers with intermittent exotropia (IXT) in China.

**Methods:**

A total of 199 IXT patients with myopia were included in this retrospective study and were divided into two groups according to the difference between near and distance exodeviation: basic IXT and convergence insufficiency (CI) IXT. Refractive errors were analyzed by spherical equivalent (SE) values. Patients were further stratified into anisometropia group and non-anisometropia group based on binocular SE values difference greater than 1.0D or not.

**Results:**

There were 127 patients in the CI IXT group, with a near deviation of 46.94 ± 20.53 prism diopters (PD) and a distance deviation of 28.36 ± 14.34 PD, and there were 72 (36.2%) patients in the basic IXT group, with a near deviation of 37.68 ± 22.21 PD and a distance deviation angle of 33.21 ± 23.96 PD. The near exodeviation was significantly larger in the CI group than in the basic IXT group(P < 0.001). In the CI IXT group, the mean SE was − 2.09 ± 1.45 diopters (D) in the dominant eye and − 2.53 ± 1.44D in the non-dominant eye, while in the basic IXT group, the mean SE was − 2.46 ± 1.56D in the dominant eye and − 2.89 ± 1.37D in the non-dominant eye. The anisometropia group included 43 patients, while non-anisometropia group included 156 patients. The near and distance exodeviation in the anisometropia group were 45.26 ± 24.41 PD and 33.53 ± 23.31 PD, respectively, and those in the non-anisometropia group were 43.42 ± 20.69 PD and 29.07 ± 16.84 PD, respectively. There were no significant differences in near and distance deviation (P = 0.78 and P = 0.73 respectively) between the two groups. The SE of the dominant eye was less myopic than of the non-dominant eyes in both the CI and anisometropia groups (P = 0.002 and P < 0.001, respectively).

**Conclusions:**

Our study revealed that convergence insufficiency IXT is more common than the basic type in pediatric myopic population and is characterized by higher inter-eye differences of myopia. The dominant eye was found to be less myopic in IXT patients, particularly in those with convergence insufficiency and anisometropia.

## Introduction

Intermittent exotropia (IXT) is the most common type of exotropia, accounting for approximately 25% of strabismus cases in children in the Western world and 44.9% in children in China [1, 2]. In children with IXT, emmetropia and myopia are the most common refractive errors [3]. There is a high prevalence of IXT and an even higher prevalence of myopia in Asia, prompting several studies to explore the relationship between these two conditions. A meta-analysis reported that children with myopia had a 5.23-fold increased risk of developing exotropia [4]. Another population-based observational study indicated that children with intermittent exotropia showed a significant trend toward myopia over time [5].

Research on ocular dominance and myopia in healthy individuals has been conducted [6–9], which has been suggested that eye dominance may play a role in the development of myopia. Ocular dominance refers to a preference for the visual input from one eye when viewing binocularly. Assessing ocular dominance or fixation preference is important in managing IXT, particularly when choosing the eyes to be operated on [10]. However, research on ocular dominance and IXT patients, particularly in the pediatric myopia population, has been limited [11].

The current study aimd to compare the degree of myopia between the dominant and non-dominant eyes in teenagers with IXT in China. These findings may help to further our understanding of the relationship between myopia and IXT.

## Methods

### Study population and clinical measures

This retrospective observational study was approved by the institutional Review Board of Peking University People’s Hospital and was conducted in accordance with the tenets of the Declaration of Helsinki. The medical records of children aged 5–18 years at the time of their operation from September 2018 to September 2022 were retrospectively reviewed. These children had been diagnosed with IXT and myopia and had undergone unilateral or bilateral lateral muscle recession or unilateral recession-resection surgery as prescribed by the same surgeon.

The following preoperative characteristics were recorded from the patients’ charts: age at surgery, best-corrected visual acuity, cycloplegic refraction and preoperative motor alignment at distance and near. The angle of deviation was determined by the prism alternate cover technique with a penlight at both distance (6 m) and near (1/3 m).

All patients were measured after 1 h of monocular occlusion of the non-dominant eye Patients were classified as having basic IXT if the difference between distance and near deviation was within 10 prism diopters (PDs). Convergence insufficiency was defined as an IXT at near measurement at least 10 PDs greater than that at distance measurement.

Refractive errors were analyzed by spherical equivalent (SE) values. Patients with amblyopia, oblique muscle dysfunction, vertical strabismus, A–V pattern strabismus, paralytic strabismus, previous extraocular muscle surgery, nystagmus, and neurological or other medical problems were excluded from this study.

Myopia was defined as a minus refractive error with an absolute value of SE power greater than 0.50 diopters(D) [12]. Myopic anisometropia was defined as an interocular refractive error difference of greater than 1.00 D, with each eye being either emmetropic or myopic. Astigmatism in both eyes should be less than 1.50 D [13].

### Ocular dominance

Ocular dominance testing was a part of our routine practice of intermittent exotropia patients. We performed the hole-in-the-card test [14]. The patient was asked to hold a card with a hole in the middle using both hands and was told to view a 6-m target through the hole with both eyes open. Then each eye was occluded alternately by the examiner to establish which eye was aligned with the hole and the target. The process was repeated three times for confirmation.

### Statistical analysis

The statistical analysis was performed with the R programming package (version 4.2.2; The R Foundation, Vienna, Austria). Means ± standard deviations were used for continuous variables and frequencies were presented for categorical variables. We perform Chi-square test for categoric parameters. A Wilcoxon rank-sum test was used to test the significant refractive error and exodeviation differences in continuous variables between groups. P ≤ 0.05 was considered to be statistically significant.

## Results

The characteristics of the study participants are presented in Table 1. A total of 199 patients were included in this retrospective study, consisting of 95 girls and 104 boys, with a mean age of 9.95 ± 2.73 years. The average near and distance deviation was 43.59 ± 21.57 PD and 30.12 ± 18.50 PD, respectively, which has significant difference(P < 0.001). The average SE of the non-dominant eye was − 2.67 ± 1.42D, and the average SE of the dominant eye was − 2.25 ± 1.47D, with a significant difference(P = 0.007).

**Table 1:** Demographic characteristics for intermittent exotropia (IXT) patients

	Total	CI	Basic	P value
N	199	127	72	
Boys:Girls(n)	104:95	69:58	35:37	0.71
Age(year)*	9.95 ± 2.73	9.93 ± 2.41	10.00 ± 3.24	0.77
Near Deviation (PD) ^*^	43.59 ± 21.57	46.94 ± 20.53	37.68 ± 22.21	< 0.001
Distance Deviation (PD) ^*^	30.12 ± 18.50	28.36 ± 14.34	33.21 ± 23.96	0.93
SE of right eye(D) ^*^	-2.50 ± 1.45	-2.41 ± 1.45	-2.72 ± 1.43	0.08
SE of left eye(D) ^*^	-2.25 ± 1.50	-2.21 ± 1.47	-2.64 ± 1.54	0.03
Dominant-eye (Right: Left)(n)	95: 104	61:67	34:37	0.55
SE of dominant eye(D) ^*^	-2.25 ± 1.47	-2.09 ± 1.45	-2.46 ± 1.56	0.07
SE of non-dominant eye (D) ^*^	-2.67 ± 1.42	-2.53 ± 1.44	-2.89 ± 1.37	0.05

CI: convergence insufficiency

PD: prism diopters

SE: spherical equivalent

*:data was shown in means ± standard deviation

Out of the 199 patients, 127 (63.8%) were classified in the convergence insufficiency (CI) IXT group, with a mean near deviation of 46.94 ± 20.53 PD and mean distance deviation of 28.36 ± 14.34 PD, while 72 (36.2%) were in the basic IXT group, with a mean near deviation of 37.68 ± 22.21 PD and mean distance deviation of 33.21 ± 23.96 PD. In the CI IXT group, the mean SE was − 2.09 ± 1.45D in the dominant eye and − 2.53 ± 1.44D in the non-dominant eye, while in the basic IXT group, the mean SE was − 2.46 ± 1.56D in the dominant eye and − 2.89 ± 1.37D in the non-dominant eye. The near exodeviation was significantly larger in the CI group than in the basic IXT group(P < 0.001), and the average SE of the non-dominant eye was slightly more myopic in the basic group than in the CI group(P = 0.05). There were no differences in gender, age, distance deviation, or SE of the dominant eye between the CI and basic groups.

According to the binocular SE differences, the patients were divided into the anisometropia group and non-anisometropia group, as shown in Table 2. The anisometropia group included 43(21.6%) patients, with a mean age of 11.54±2.50 years, and non-anisometropia group included 156(78.4%) patients, with a mean age of 9.52±2.64 years, showing that patients in the anisometropia group were older (P < 0.001). The near and distance exodeviation in the anisometropia group were 45.26 ± 24.41 PD and 33.53 ± 23.31 PD, respectively, and those in the non-anisometropia group were 43.42 ± 20.69 PD and 29.07 ± 16.84 PD, respectively,. There were no significant differences in near and distance deviation between the two groups (P = 0.78 and P = 0.73 respectively). Additionally, near exodeviation was significantly greater than distance exodeviation both in anisometropia group and non-anisometropia group (P = 0.004 and P < 0.001, respectively). The SE of the dominant eye was less myopic in the anisometropia group, as compared with the non-anisometropia group (-1.20 ± 1.50D vs. -2.42 ± 1.39D, P < 0.001), and the SE of the non-dominant eye was more myopic in the anisometropia group (-3.08 ± 1.38D vs. -2.59 ± 1.36D, P = 0.038).

**Table 2:** Characteristics of patients in the anisometropia and non-anisometropia group

	Anisometropia	Non-anisometropia	P-Value
N	43	156	
Boys:Girls(n)	20: 23	84: 72	0.17
Age(year)*	11.54 ± 2.50	9.52 ± 2.64	< 0.001
Near Deviation (PD) ^*^	45.26 ± 24.41	43.42 ± 20.69	0.78
Distance Deviation (PD) ^*^	33.53 ± 23.31	29.07 ± 16.84	0.73
IXT group			0.48
CI, n(%)	25(58.1)	102(65.4)	
Basic, n(%)	18 (41.9)	54(34.6)	
SE of right eye(D) ^*^	-2.58 ± 1.58	-2.51 ± 1.41	0.90
SE of left eye(D) ^*^	-1.93 ± 1.84	-2.48 ± 1.38	0.008
Dominant-eye (Right: Left)(n)	19:24	76:80	
SE of dominant eye(D) ^*^	-1.43 ± 1.58	-2.44 ± 1.48	< 0.001
SE of non-dominant eye (D) ^*^	-3.08 ± 1.38	-2.59 ± 1.36	0.038

CI: convergence insufficiency

PD: prism diopters

SE: spherical equivalent

*:data was shown in means ± standard deviation

As shown in Table 3, the SE of the dominant eye was significantly lower than that of the non-dominant eye in all participants (P = 0.007), particularly in the CI group and anisometropia group (P = 0.002 and P < 0.001, respectively). Right eyes were also significantly more myopic than the left eyes in the anisometropic IXT patients (P = 0.03).

**Table 3:** Spherical equivalent of eyes in IXT patients

	SE of dominant eye (mean ± SD)	SE of non-dominant eye (mean ± SD)	P value	SE of Right eye (mean ± SD)	SE of Left eye (mean ± SD)	P value
Total	-2.25 ± 1.47	-2.67 ± 1.42	0.007	-2.50 ± 1.45	-2.25 ± 1.50	0.17
CI	-2.09 ± 1.45	-2.53 ± 1.44	0.002	-2.41 ± 1.45	-2.21 ± 1.47	0.16
Basic	-2.46 ± 1.56	-2.89 ± 1.37	0.08	-2.72 ± 1.43	-2.64 ± 1.54	0.70
Anisometropia	-1.43 ± 1.58	-3.08 ± 1.38	< 0.001	-2.58 ± 1.58	-1.93 ± 1.84	0.03
Non-Anisometropia	-2.44 ± 1.48	-2.59 ± 1.36	0.32	-2.51 ± 1.41	-2.48 ± 1.38	0.73

CI: convergence insufficiency

SE: spherical equivalent

SD: standard deviation

## Discussion

According to the Burian’s classification, IXT could be divided into basic, divergence excess, pseudo-divergence excess, and convergence insufficiency [15]. The convergence insufficiency type, characterized by near deviation exceeding the distance deviation by at least 10 PD, is estimated to account for 1.2–11% of IXT cases [16, 17]. Our study found a higher proportion of convergence insufficiency type IXT patients (63.8%), which may be because all of the patients in our study had myopia. Myopic children showed greater divergent shift in vergence adaptation after near work [18]. It is speculated that in myopic patients, fusional control at distant of myopes is weakened due to the blurred distant vision, and for near vision, less accommodative effort is required for clear images in myopes due to a larger accommodation lag [19], which may lead to reduced accommodative convergence and ultimately result in convergence insufficiency [20].

The refractive error of the basic IXT group was more myopic than that in the CI IXT group. Patients with CI IXT typically have a reduced near point of convergence (NPC) and positive fusional vergence (PFV) for near viewing, as well as a low accommodative convergence to accommodation (AC/A) ratio [21]. However, higher AC/A ratios was related to a greater lag of accommodation and have been reported as a risk factor for myopia in children [22, 23].

As shown in Table 3, our results demonstrated that SE of the dominant eye was significantly less myopic than that of the non-dominant eye in the CI group(P = 0.002), but not in the basic group(P = 0.08), indicating that CI IXT patients have a greater difference in SE of the two eyes. Yang and Hwang [24] found that IXT patients displayed a greater lag of accommodation in the non-dominant eye compared to the dominant eye during binocular fixation, which provides a potential mechanism for why the nondominant eye becoming more myopic [25]. Patients with IXT suffer a large accommodative load, which has been proven to be related to the degree of exodeviation [25]. CI IXT patients exhibit a larger near-distance deviation difference than basic IXT, which might require greater accommodation at near viewing, leading to greater interocular asymmetry.

Furthermore, we divided IXT patients into the anisometropia and non-anisometropia groups based on intraocular refractive difference. Participants with anisometropia were much older, and previous studies have indicated that the prevalence and magnitude of anisometropia would increase in association with age throughout childhood to young adulthood [13, 26]. Our study revealed that the SE of the dominant eye was less myopic in the anisometropia group, while the SE of the non-dominant eye was more myopic. Additionally, the right eyes were more myopic in children with anisometropia. Linked et al. [6]. reported that the nondominant eye is more myopic in the anisometropia patients with interocular SE differences greater than 2.5 D. Wang et al. [27] identified the nondominant eye had a tendency of higher refraction when SE anisometropia larger than 1.75 D in adults. However, the results on which eye is more myopic are conflicting, possibly due to the discrepancies in subject ethnicity, age, magnitude of anisometropia and different methods for measuring ocular dominance [8, 13, 28].

There are several limitations to this study. It was a retrospective design study with a relatively small number of patients, which may introduce bias. Moreover, this study is cross-sectional, so it is not clear whether the difference in refractive error between the two eyes is a result of IXT or an adaptation. Longitudinal studies are needed to fully understand the relationship between IXT and ocular dominance refractive status. A longitudinal study is currently being conducted in our lab to examine whether ocular sensory dominance changes during the progression of IXT.

In conclusion, our study revealed that the convergence insufficiency type is more common than the basic type in Chinese myopic children and adolescents, and it is characterized by a higher inter-eye difference of myopia. The dominant eye was found to be less myopic in IXT, particularly in those with convergence insufficiency and anisometropia. Further research is needed to fully understand the relationship between ocular dominance and myopia in IXT patients.

## Data Availability

The datasets used and/or analyzed during the current study are available from the corresponding author on reasonable request.

## Abbreviations

IXT: Intermittent exotropia
CI: Convergence insufficiency
SE: Spherical equivalent
PD: Prism diopters
